# Mid-infrared Ring Interband Cascade Laser: Operation at the Standard Quantum Limit

**DOI:** 10.1021/acsphotonics.3c01159

**Published:** 2024-01-18

**Authors:** Georg Marschick, Jacopo Pelini, Tecla Gabbrielli, Francesco Cappelli, Robert Weih, Hedwig Knötig, Johannes Koeth, Sven Höfling, Paolo De Natale, Gottfried Strasser, Simone Borri, Borislav Hinkov

**Affiliations:** † TU Wien—Institute of Solid State Electronics & Center for Micro- and Nanostructures, Gußhausstraße 25-25a, Vienna 1040, Austria; ‡ University of Naples Federico II, Corso Umberto I 40, Napoli 80138, Italy; § CNR-INO—Istituto Nazionale di Ottica, Largo Fermi, 6, Firenze, FI 50125, Italy; ∥ CNR-INO—Istituto Nazionale di Ottica, Via Carrara, 1, Sesto Fiorentino, Florence 50019, Italy; ⊥ LENS—European Laboratory for Non-Linear Spectroscopy, Via Carrara, 1, Sesto Fiorentino, Florence 50019, Italy; # nanoplus Nanosystems and Technologies GmbH, Oberer Kirschberg 4, Gerbrunn 97218, Germany; ¶ Julius-Maximilians-Universität Würzburg—Physikalisches Institut, Lehrstuhl für Technische Physik, Am Hubland, Würzburg 97074, Germany; ∇ INFN—Istituto Nazionale di Fisica Nucleare, Via Sansone, 1, Sesto Fiorentino, Florence 50019, Italy

**Keywords:** mid-infrared, optoelectronics, interband cascade laser, balanced detection, intensity noise, shot-noise, quantum limit

## Abstract

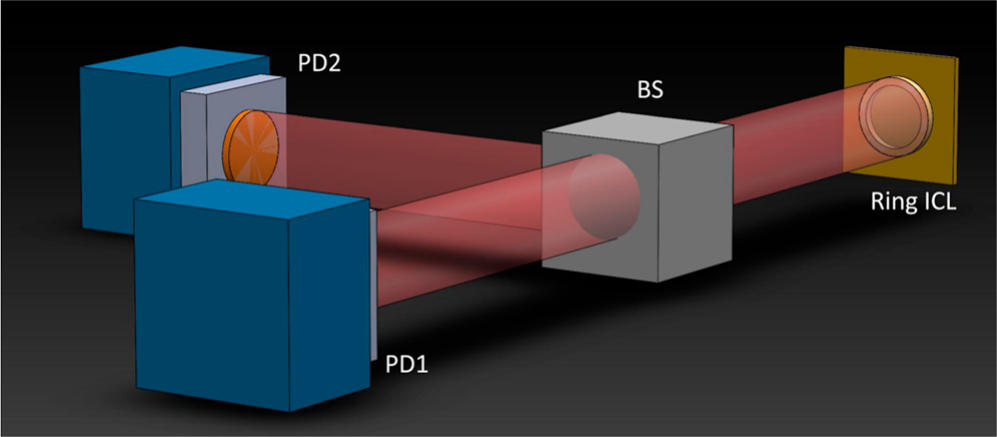

Many precision applications in the mid-infrared spectral range have strong constraints based on quantum effects that are expressed in particular noise characteristics. They limit, e.g., sensitivity and resolution of mid-infrared imaging and spectroscopic systems as well as the bit-error rate in optical free-space communication. Interband cascade lasers (ICLs) are a class of mid-infrared lasers exploiting interband transitions in type-II band alignment geometry. They are currently gaining significant importance for mid-infrared applications from < 3 to > 6 μm wavelength, enabled by novel types of high-performance ICLs such as ring-cavity devices. Their noise behavior is an important feature that still needs to be thoroughly analyzed, including its potential reduction with respect to the shot-noise limit. In this work, we provide a comprehensive characterization of λ = 3.8 μm-emitting, continuous-wave ring ICLs operating at room temperature. It is based on an in-depth study of their main physical intensity noise features such as their bias-dependent intensity noise power spectral density and relative intensity noise. We obtained shot-noise-limited statistics for Fourier frequencies above 100 kHz. This is an important result for precision applications, e.g., interferometry or advanced spectroscopy, which benefit from exploiting the advantage of using such a shot-noise-limited source, enhancing the setup sensitivity. Moreover, it is an important feature for novel quantum optics schemes, including testing specific light states below the shot-noise level, such as squeezed states.

## Introduction

Interband cascade lasers (ICLs) are semiconductor-based, coherent mid-IR light sources, first demonstrated by Yang et al. in 1995.^1^ They are the interband counterpart to quantum cascade lasers (QCLs), which instead rely on intersubband transitions,^2^ and have been the dominant mid-IR lasers since their realization in 1994.^3^ These sources have immediately attracted wide interest in view of the many potential applications, with a focus on molecular species detection in solid,^4,5^ liquid,^6,7^ and gas phases.^8,9^ This has sparked, e.g., important works in greenhouse gas detection of methane, carbon dioxide, or nitrous oxide, including the detection of the most elusive gas isotopes,^10^ in high-sensitivity gas measurements down to the ppq level^11,12^ even in real-world applications, or in broadband (>10 cm^–1^), high-resolution (MHz-range) spectroscopy techniques like dual-comb spectroscopy.^13^ Moreover, other important mid-IR applications are currently getting significant attention, such as spectral imaging^4,5,14^ and, in more recent years, optical free-space communication.^15−17^

This large interest acted as a strong driving force for the technological development of these sources. ICLs differ from QCLs, for example, by their much lower power consumption and their operation at shorter wavelengths, even below 3 μm. Due to these and other peculiarities, ICLs are nowadays in many fields competitive with their QCL counterparts, matching the requests for high optical output power,^18,19^ wide spectral tunability,^20^ comb emission,^21^ compact dimensions and integrability,^20^ spectral control and (ultra)narrow line width,^22−24^ and low noise emission.^25^

ICLs are the result of combining the strong interband transitions and long recombination lifetimes inherent to traditional diode lasers^26^ with the voltage-efficient cascading design of QCLs^3^ into an active region (AR) using type-II band alignment. This allows for maintaining the QCL-like flexibility in designing the emission wavelength of ICLs through band-structure engineering while simultaneously strongly reducing their number of AR periods. As mentioned, the result is significantly lower power consumption, e.g., at a laser threshold^27^ of around 170 mW,^23^ to compare to even specifically optimized low dissipation QCLs with threshold dissipation values between 350 and 850 mW.^28−30^ This advantage is particularly important for portable ICL-based sensors^31^ or for future space deployment. In novel ring-ICLs, ring-shaped ridge cavities are used together with vertical light emission, merging multiple advantages into a single device. First, the ring-cavity shares radial symmetry with most discrete optical elements such as lenses and mirrors, which is beneficial for light collimation or focusing. Second, the large effective surface area of circular waveguides offers a large aperture, providing small divergent emission beams with angles below ±10° and thus simpler collimation.^23^ Third, previous work in QCLs has revealed that ring geometries, due to their different mode distribution within the cavity as compared to straight ridges, offer specific, electronically controllable frequency-modulation (FM) states,^32^ which are useful features for high-speed spectroscopy.^33^ Fourth, compared to other vertical surface-emitting lasers, such as vertical-cavity surface-emitting lasers (VCSELs),^34,35^ with their limited output power due to small gain volumes, the output power of ring ICLs can be scaled up by simply increasing the ring diameter or the waveguide width. In this case, obeying certain design guidelines prevents higher-order lateral modes.^23^

For controlling line width, single-mode emission capabilities, and vertical light outcoupling in ring ICLs, distributed feedback (DFB) gratings etched into the laser waveguide which periodically modulate the complex refractive index of the waveguide can be used.^36,37^ Design and fabrication of the DFB grating are some of the most important steps in order to achieve a well-functioning device. While the grating period width Λ is determined by the Bragg condition , with *n*_eff_ being the effective refractive index, *m* describing the grating order, and λ being the design emission wavelength, the precise influence of the grating etch depth as well as the grating duty cycle is either obtained by optical simulations or determined experimentally. DFB gratings have already been successfully integrated into ICLs using various waveguide geometries.^22,23,38^ Especially in the case of vertically emitting devices, a second-order DFB grating is needed. The optical feedback necessary for single-mode operation and vertical light coupling is introduced through the second-order Bragg scattering (order *m* = 2) which simultaneously rotates the Poynting vector by ±90° and selects one single emission wavelength.^23,38^ This opens the pathway to implement 2D multiwavelength concentric array geometries,^39^ an important step toward broadband chip-scale spectrometers.

Despite all the achievements of ICLs, their intensity noise together with its potential reduction in ring ICLs still needs to be thoroughly characterized. Fundamentally, intensity noise in semiconductor lasers like ICLs originates from their various internal electronic and optical processes such as spontaneous emission and random carrier generation/recombination.^25^ Understanding its characteristics is important for increasing the sensitivity and resolution of imaging or spectroscopic systems^25,40^ and for telecommunication concepts with reduced bit-error rates.^41^ Furthermore, it is of particular relevance in the future development of quantum optics schemes, such as homodyne detection, where a shot-noise-limited source is highly desirable, as a local oscillator, to test light states below the shot-noise level (e.g., squeezed states).^42,43^

In the current work, we follow this need and investigate for the first time the relative intensity noise (RIN) of a single-mode-emitting ring-ICL. The device operates in continuous-wave (CW) mode at room temperature with an emission wavelength of λ = 3.8 μm. As previously discussed, ring devices have beneficial features for spectroscopic applications as compared to similar ridge devices.^23,32,39^ In our study, we first analyze the light-current–voltage (LIV) and single-mode emission characteristics of a typical custom-made second-order DFB ring ICL. Then, a balanced-detection setup, consisting of a 50/50 beam splitter and two identical photovoltaic detectors, is employed to characterize the intensity noise power spectral density (INPSD) of the ring ICL and compare it to the directly measured shot-noise level. We further analyze the RIN of the ICL under different laser driving conditions to understand the optimal low-intensity-noise working regime of the tested device geometry.

## Device Structure and Working Principle

**Figure 1:**
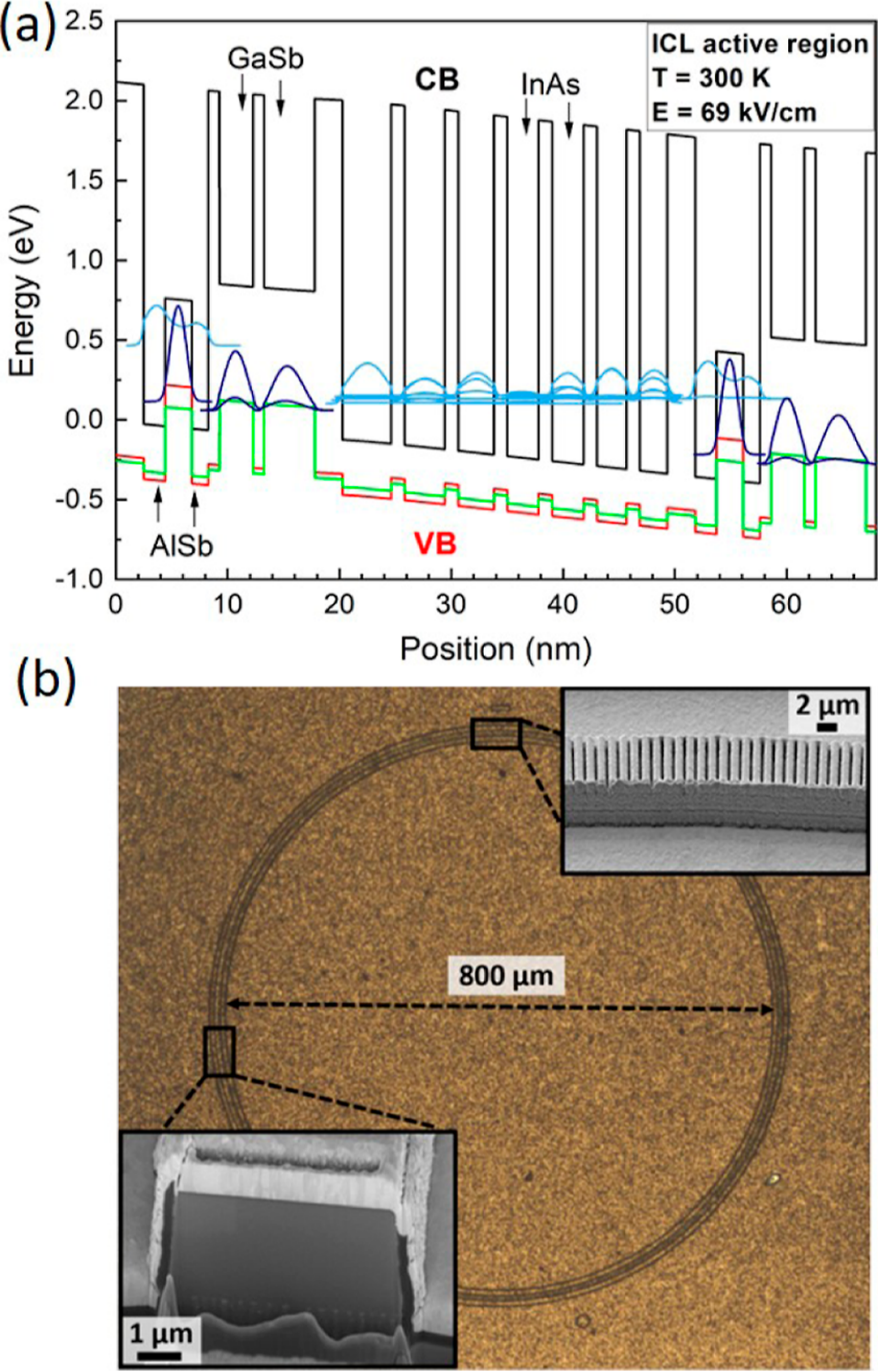
(a) Band structure of the ring ICL including the simulated wave functions for an applied field of 69 kV/cm. (b) Microscope image of the fabricated ring ICL. The insets show: (top right) a detailed view of the ring waveguide with the implemented 2nd-order DFB grating for vertical light coupling and single-mode emission and (bottom left) a SEM image of an FIB cut through the ridge of the ring device.

The quantum structure of the device investigated in this work is grown by solid-source molecular beam epitaxy (MBE) on an n-GaSb (100) substrate. The w-shaped AR with 6 periods follows the layer sequence 2.50 nm AlSb/1.92 nm InAs/2.40 nm In_0.35_Ga_0.65_Sb/1.49 nm InAs/1.0 nm AlSb for a target emission wavelength of 3.8 μm. It is sandwiched between two 200 nm thick GaSb separate confinement layers as well as a 3.5 and 2.0 μm InAs/AlSb lower and upper cladding, respectively. Since within the superlattice-like structure of the active region, most interfaces involve a change of both group III and group V materials, careful optimizations of interface roughness and strain balance were carried out using so-called group V soak times during the growth. Figure 1a shows the band structure including simulated wave functions of the AR design for an applied external field of 69 kV/cm. The epitaxial ICL structure is processed into ring-shaped cavities with a diameter of ∼800 μm and a ridge width of 6 μm (circumference: approximately 2.5 mm) using state-of-the-art cleanroom fabrication techniques. Special attention is given to the below-1 micrometer feature size of the implemented second-order DFB grating for vertical and single-mode light coupling, which was patterned through electron-beam lithography and as a next step etched around 1100 nm deep into the already existing waveguide with Cl2–Ar reactive ion etching. The structure of the device characterized in this work is similar to the device characterized in ref (23) with the mentioned main differences of a wider waveguide (6 μm instead of 5 μm) and a different DFB grating period to address shorter wavelengths. The measured device is a typical representative of the processed ring ICLs; thus, the results shown in this work are expected to be a benchmark for the noise behavior of devices with similar epi-structure and dimensions. Figure 1b displays a microscope image of a typical finalized ring-ICL device, including scanning electron microscopy (SEM) images of the DFB grating implemented on the laser waveguide and a focused ion beam (FIB) cut through the ridge of the ring device revealing its high-quality cross-section profile.

Substrate-side emission is the preferred geometry for such devices, which allows covering the entire topside of the rings including the DFB grating structure with gold and the use of flip-chip bonding on copper submounts with indium solder. This results in significantly improved heat extraction from the device AR and is important for a high-performance CW operation. More details on the AR design and device fabrication can be found elsewhere.^23^

## Device Characterization

### LIV Curve and Emission Spectrum Characterization

**Figure 2:**
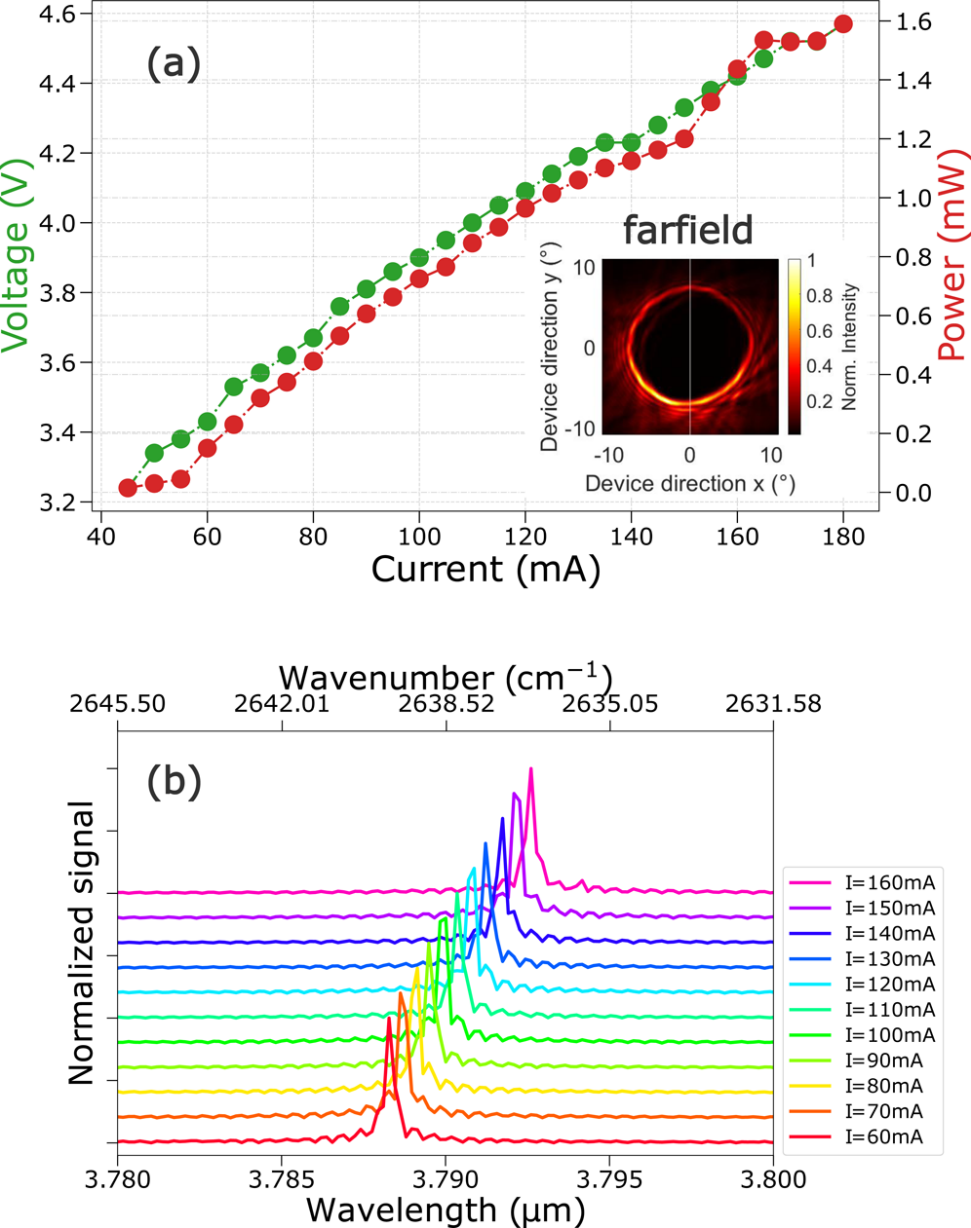
Ring-ICL characterization at a fixed temperature of 16 °C. (a) LIV curve of the ring ICL analyzed between ∼40 and 180 mA. The measured optical power is shown in red and the associated voltage in green. Inset: far-field measured with a HgCdTe-detector on a *xy*-stage and at a distance of 20 cm to the ring-ICL device. (b) Corresponding, individually normalized, bias-dependent emission spectra of the ring ICL measured with an optical spectrum analyzer (FTIR 721, Bristol), which has a resolution of 6 GHz (i.e., 0.2 cm^–1^). The y-scale is linear, and the tick span is 0.5 au. The emission spectra show an SMSR up to 30 dB as compared to the flat part of the spectrum away from the peaks. It is worth noting that the traces have been normalized to their respective maximum peak and an offset is added in the *y*-direction to allow a good visibility of all the spectra within a single graph [In brief, the SMSR was computed by dividing the peak value by the mean level of the spectrum calculated far from the peaks (around 3.85 μm), which is limited by the instrumental background, and has then been translated to the dB scale. The maximum SMSR value, i.e., approximately 30 dB, refers to the maximum peak signal recorded in the whole series of FTIR acquisitions (i.e., for bias currents of 140–160 mA). Since the peak maximum value changes for each acquisition at different operating currents, in Figure 2, the acquired traces have been normalized to their individual peak value to allow a clear view of the emitted spectra as a function of the applied bias current within a single plot.].

First, a typical ring ICL is characterized in order to determine its optimal working point when operated at a fixed temperature of 16 °C in CW mode [The temperature of 16 °C for the characterization was chosen to simultaneously satisfy: (1) a high enough optical output power of the laser (for which a lower temperature is beneficial) together with (2) laser operation that does not need a more sophisticated setup including, e.g., purging of the laser with dry air/nitrogen to prevent water condensation at the laser facet from ambient humidity.]. In our setup, the ring ICL is driven by an integrated modular controller (ppqSense s.r.l., QubeCL10) including temperature stabilization by using a thermo-electric cooler. Its laser driving unit is characterized by a low bias current noise density, typically around 300 pA/, for reducing its effect on the intensity noise of the operated device. As shown by the LIV curve in Figure 2a, the tested ring ICL exhibits a lasing threshold of around 50 mA when operated at 16 °C, while it reaches its maximum optical output power of approximately 1.6 mW at 160 mA. Regarding the measured optical spectra shown in Figure 2b, the laser maintains a well-defined single-mode emission at around 3.79 μm within its whole working range, reaching a side-mode suppression ratio (SMSR) of up to 30 dB. As expected, the emission peak moves to longer wavelengths with increasing laser bias current, going from λ = 3.788 μm at 60 mA to λ = 3.793 μm at 160 mA. By analyzing the laser peak emission wavelength as a function of bias current, we obtain a current-tuning coefficient of  MHz/mA. More details regarding this analysis are presented in Appendix A in the Supporting Information.

While the optical emission power of this specific ring ICL is limited, especially when compared to typical mid-IR QCLs or ICLs, both of which can reach emission powers of tens to hundreds of milliwatts,^27,44−46^ our ring device is able to operate at very low consumed electrical power (at maximum bias: ∼160 mA at ∼4.5 V). This demonstrates its suitability for in-field applications where energy resources are limited to battery operation or even solar energy only.^47,48^ Moreover, an optical emission power of about 1 mW is often sufficient for sensing applications,^26^ as long as the target wavelength is precisely hit. Indeed, depending on the detector sensitivity, hundreds of microwatts of optical power can be sufficient for transmission spectroscopy applications also,^49^ as well as novel chip-level applications using compact photonic integrated circuits.^50,51^ We therefore focused our work on the spectral stability and purity of the laser emission for achieving low-noise characteristics instead of trying to improve “traditional” figures of merit such as the optical emission power or wall-plug efficiency. Thus, the high spectral purity and stability of our ring ICL can be considered suitable for different state-of-the-art applications, including cavity-enhanced spectroscopy experiments,^52−55^ free-space optical communication,^56^ and metrological measurements.^57^

To finalize the basic optical characterization of the ring ICL, we report, in the inset of Figure 2, the acquired far-field profile of the device, measured with an MCT detector mounted on a translational xy-stage which was placed at a distance of 20 cm from the ring ICL. The ring-shaped geometry with its typical dark central part and a narrow circular beam hosting the device power can clearly be observed.

### Intensity Noise Characterization Using Balanced Detection

**Figure 3:**
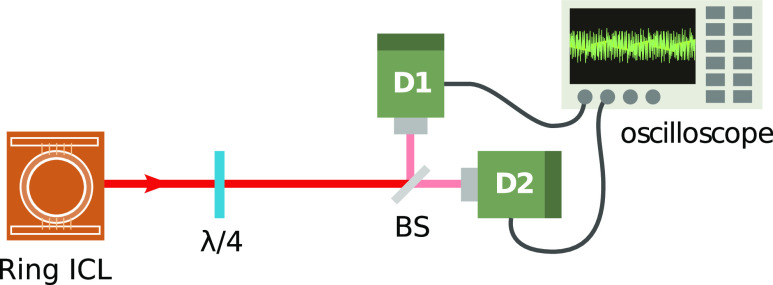
Schematic representation of the experimental setup used for balanced detection. The figure is readapted from ref (43).

In order to understand the intensity noise features of the presented ring ICL, we analyzed its INPSD using a balanced-detection experiment. In this setup, sketched in Figure 3,^43^ the light under investigation is split into two identical beams via a 50/50 beam splitter and acquired via two commercial HgCdTe photovoltaic detectors (D1 and D2) equipped with a 5 MHz-bandwidth preamplifier (VIGO Photonics S.A., amplifier: PIP-UC-LS, detector: PV-4TE-4-1 × 1). The used MCT detectors are optimized for having a high saturation level, that is, about 12 mW. They have been chosen for minimizing the “optical attenuation” required for performing the measurements since the introduction of additional optical attenuation alters the original ratio between the observed intensity noise and the related computed shot noise. The saturation level is so high because they are large-area detectors; therefore, they cannot have a fast response.

The electronic architecture of the detectors consists of two amplifier stages: a 6 kΩ preamplifier transimpedance stage, where the dc-output is collected, and a second stage for which coupling (i.e., ac or dc) and gain can be chosen via a PC software (VIGO Photonics S.A., Smart Manager). In our case, the ac-coupled second stage is used to amplify the ac-voltage noise amplitude by a factor of 62 V/V [we fixed the gain to 20 via the Smart Manager control platform, but we also measured a minimum amplification amount between the 1st-stage output voltage and the 2nd-stage output voltage equal to 3.1 V/V when the ac gain is set to 1. The two factors (3.1 × 20 in our case) need to be multiplied to retrieve the actual voltage amplification factor.]. The detectors are maintained at a fixed temperature of 200 K by a four-stage Peltier cooling system using a thermometric cooler controller (VIGO Photonics S.A., PTCC-01-BAS). The signals are analyzed in the time domain. In particular, a 12 bit oscilloscope (Tektronix, MSO64) is used to acquire the two detectors’ output signals in a 20 ms time window and at a fixed sampling rate of 31.25 Ms/s. In our measurements, the oscilloscope bandwidth is limited to 20 MHz. Finally, a Python script is used to compute the sum and the difference of the acquired signal and to convert them from the time to the frequency domain, computing the INPSD of the difference and of the sum.^43^ Since at each point, the polarization is tangential to the beam (i.e., circular), a λ/4 wave plate is placed just after the device to retrieve a linear polarization (a λ/2 wave plate is also needed to equally balance the two split arms.). The emitted laser beam from the chip is uncollimated; therefore, we placed an additional 50 mm lens in front of each detector to collect all the light within its 1 × 1 mm^2^ collection area.

For evaluating the performance of the assembled balanced-detection system, we performed a preliminary characterization of the used photodetectors. One key parameter in our measurements is the detector responsivity, defined as the detector output signal (voltage or current) as a function of the incident optical power. In particular, in order to perform a balanced detection in which the common noise of both arms is suppressed at the shot-noise level, it is necessary to use two photovoltaic detectors with a responsivity that is as similar as possible. Otherwise, the detection is unbalanced in favor of one of the two arms, even when investigating two initially identical incident optical signals on D1 and D2. Thus, when performing the balanced-detection experiments in the linear responsivity regime of two detectors, the INPSD computed from the difference of the photocurrent output signals is expected to be at the shot-noise level and, therefore, proportional to the incident power impinging on the beam splitter.^43^ This is true in the limit given by the maximum common mode rejection ratio (CMRR) achievable with our setup, i.e., the maximum excess of noise with respect to the shot-noise level that can be canceled with our differential measurement.^43^ Instead, the sum of the two photocurrent ac output signals corresponds to the measurement of the whole intensity noise associated with the radiation impinging on the balanced detector. It is linked to the intensity noise of the laser minus a possible attenuation factor (due to the losses experienced by the propagating beam and the detector efficiency), plus an extra contribution due to the coupling of the tested radiation with the vacuum field caused by the losses^43^ [from a quantum optics point of view, “coupling the radiation with the vacuum field” means that via optical attenuation (e.g., attenuation due to optical elements such as lenses, isolators, and beam splitters, quantum efficiency of the used detectors, and absorption of the laser waveguide), the radiation is attenuated and the statistics of the photon flux is altered toward a Poissonian distribution. In quantum optics, the attenuations are modeled with beam splitters, where one input port is used for the signal, while the other has no field except the vacuum. This is why it is said that the losses couple the radiation to the vacuum.]. Under the condition of balanced detection performed in the linear responsivity regime and assuming the noise level does not exceed the maximum CMRR, it is sufficient to directly compare the retrieved INPSD of sum and difference for judging whether the light collected from the source under investigation is shot-noise-limited. This means that its photons are Poissonian-like distributed, as expected for a coherent light source.^42^ Based on these considerations, we carefully selected two photodetectors with a very similar responsivity at λ = 3.79 μm of *R*_1_ = (0.704 ± 0.007) A/W and *R*_2_ = (0.659 ± 0.008) A/W, respectively. Furthermore, when the differential measurement is performed, a CMRR of up to 25 dB is achievable in the tested bandwidth. More details of this analysis are available in Appendix C in the Supporting Information.

**Figure 4:**
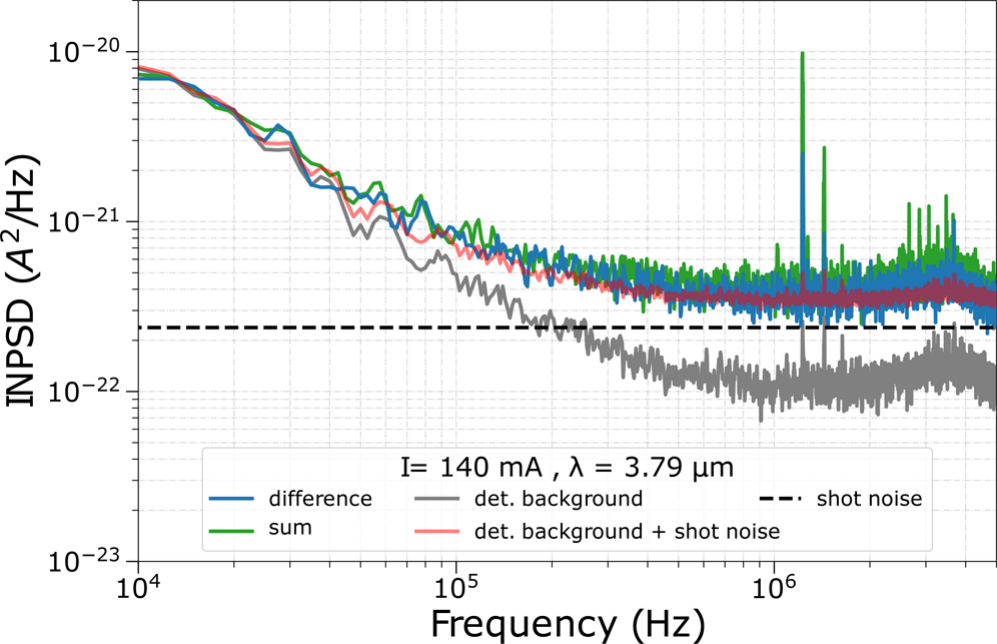
Ring-ICL INPSD analysis performed at a fixed temperature of 16 °C and at a laser bias current of 140 mA. The INPSD sum and difference signal traces are colored green and blue, respectively. The dashed black line represents the theoretical shot-noise level, obtained from the dc outputs of the detectors, while the detector background is shown in gray. The red line shows the sum of the shot-noise level and detector background noise. In the INPSD of the detector background and of the laser, some spurious noise peaks at slightly above 1 MHz are present. They are due to technical noise originating from different sources including intrinsic electronic noise of the current driver, mass-loop noise due to its power supply, and the supply used for the detectors. This technical noise can be reduced, e.g., by using battery operation. It is important to note that even though these peaks are present, still a shot-noise-limited intensity noise for the tested ICL is demonstrated, with the exception of those few particular frequencies.

Figure 4 shows the INPSD of the ring ICL analyzed at 140 mA, which corresponds to a condition in which the laser is affected neither by noise contributions from spontaneous emission events close to the laser threshold nor by any saturation effects close to the device rollover, as shown in Figure 2. The output power under these driving conditions is around 1.2 mW. Therefore, the detectors, each receiving around 0.6 mW, are not saturated (the optical losses due to the optical tools, e.g., mirrors, wave plates, and lenses are around 2%). As evidenced in Figure 4, the INPSD of the difference signal (blue trace) corresponds to a direct measurement of the shot-noise level: indeed, the INPSD of the difference signal overlaps with the red trace, which shows the sum of the background noise (gray trace) and the theoretically computed shot-noise power spectral density (PSD) (dashed black line). To retrieve this latter quantity, we measure the dc output of the two photovoltaic detectors and calculate the shot-noise PSD as PSD_SN_ = 2*e*(*V*_1_ + *V*_2_)/*R*, where *e* is the electron charge, *V*_1 and 2_ are the voltages measured at the two first-stage transimpedance dc-outputs of both detectors, and *R* is the transimpedance resistance value. Instead, the detector background noise was measured by blocking the laser emission via an opaque obstacle placed close to the laser (far from the detector). The signal from the detectors was therefore acquired without the contribution of laser emission. The red trace is then displayed as the sum of the gray and dashed black trace to take into account the effect of the background with respect to the calculated shot-noise level. It is important to note that, despite a non-negligible contribution of the background in the measured shot noise, the INPSD of the difference signal lies well above the sole detector background level, reaching a so-called clearance, defined as the ratio between the INPSD of the difference signal and the detector background, of up to 6 dB at a Fourier frequency of about 1 MHz.^43^ This result confirms the possibility of performing shot-noise-limited detection with the assembled setup, e.g., the setup can be successfully applied in a homodyne detection scheme using the tested ring ICL as a local oscillator.^43^ With this purpose, the optimal working conditions are those which guarantee exploitation of clearance as high as possible to minimize the effect of the background on the measurement and thus potentially increase the possibility of exploring subshot-noise signal levels in balanced detection.^42,43^ In our case, the best working conditions are therefore the use of the ring ICL at a driving current of 140 mA where it emits a power of > 1 mW which allows reaching the best clearance (i.e., 6 dB) with the assembled setup. In view of possible nonclassical application, one major limitation arises from the limited quantum efficiency of the detectors (i.e., the number of generated electrons in a detector as a function of the number of impinging photons). As shown in Appendix B of the Supporting Information, this quantity lies at around 22–23% at the investigated wavelength. Still, the results presented here give a good starting point for the development of future quantum technology systems based on the light source tested in this work. Next, we will seek to implement commercial detectors, optimized for working in the 4 μm window, with higher quantum efficiency, to potentially address quantum optics applications, where losses directly correspond to a degradation of the nonclassicality of a tested nonclassical signal (e.g., a squeezed state of light characterized by subshot-noise-level amplitude noise). This is done by mixing it with the vacuum state of the electromagnetic field for a percentage corresponding to the amount of the losses.^42,43^

Coming back to the characterization of the tested ICL, it clearly benefits from a shot-noise-limited intensity noise within the tested detector efficiency. Indeed, the obtained data show an INPSD of the sum signal (green trace, Figure 4) that is superimposed with the INPSD of the difference trace in blue for the entire investigated Fourier frequency domain. In Appendix C of the Supporting Information, we also demonstrate that this interesting behavior is similar for different laser drive currents, at a fixed laser temperature. The shot-noise-limited operation represents an important feature in ring ICLs for applications requiring a well-suppressed-intensity-noise light source, such as in quantum homodyne detection,^42,43^ high-sensitivity interferometry,^58,59^ and spectroscopy.^60^ With this purpose, it is worth noting that at lower frequencies (up to 100 kHz), all traces are background-noise-limited. Therefore, in view of future applications, the optimum working range for our balanced-detection setup is in the frequency range between 100 kHz and 5 MHz, where there is a rollover due to the limited bandwidth of the detectors.

**Figure 5:**
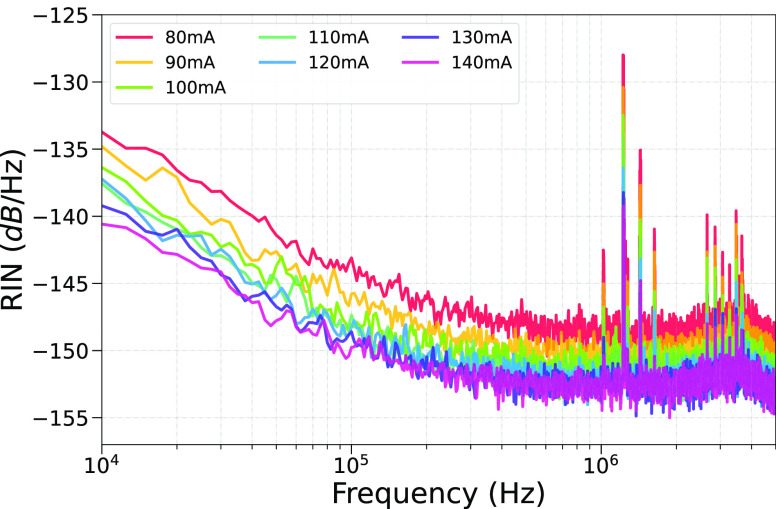
RIN of the ring ICL measured for different bias currents at a fixed temperature of 16 °C. As already discussed in Figure 4, the here-reported RIN also shows some spurious noise peaks due to the presence of excess technical noise, originating from the laser driving unit.

Finally, Figure 5 depicts the RIN of the ring ICL at different bias currents for a fixed temperature of 16 °C. The RIN is defined as the INPSD of the sum signal normalized to the square of the sum of the photocurrents measured by the two photodetectors. As expected, the RIN decreases with increasing laser bias current for measurements between *I* = 80 mA and *I* = 140 mA.

Compared to previous studies where the reported RIN levels for ICLs cover a range from −110 dB/Hz^61^ up to −130 dB/Hz,^25^ our device shows better intensity noise performance in terms of RINs which decreases down to −153 dB/Hz at a current of 140 mA. This is similar to the values found in reference 62^62^ where the tested ICL has an RIN which decreases down to −160 dB/Hz, however, at a temperature of 30 K. Fundamental differences of our devices compared to the devices from the literature include single-mode emission through the implemented second-order DFB grating and a ring-cavity geometry, which inherently supports a different internal mode structure as compared to standard Fabry–Pérot ridge devices.

## Conclusions

In conclusion, we investigated the noise characteristics of a second-order DFB ring ICL emitting at λ = 3.79 μm at a fixed temperature of 16 °C. The INPSD level found at a driving current of I = 140 mA, i.e., far from the laser threshold and from the laser rollover, with a balanced-detection setup, demonstrates shot-noise-limited operation between 100 kHz and 5 MHz. In the setup, we employed two HgCdTe photovoltaic detectors with similar responsivity, which are moreover linear over the whole range of investigated laser bias currents. Subshot-noise detection is shown to be potentially possible with such a configuration. For this purpose, the detector quantum efficiency should be improved, in order to enhance the chance of unveiling subclassical emission, by limiting losses.

We further investigated the RIN of our experimental configuration, obtaining decreasing RIN values with increasing laser bias currents. Moreover, in contrast to previous RIN studies performed with ICLs operated at room temperature^25,61^ where RIN levels up to −130 dB/Hz are reported, we show that our ring-DFB laser exhibits orders of magnitude of lower values for all the tested bias currents reaching a level down to −153 dB/Hz, approximately, which is in line with the values observed with ICLs operated at lower temperatures (100 and 30 K).^62^

In the future, better detection technology with significantly higher quantum efficiencies (currently ∼22%) is needed, to explore the subclassical regime and quantum optics applications.

## Data Availability

The data that support the findings of this study are available from the corresponding author upon reasonable request.

## Supporting Information Available

Characterization of the tuning coefficient, detector responsivities, detector CMRR, and INPSD at different laser currents (PDF)
