# Supplementary material for: Mid-infrared Ring Interband Cascade Laser: Operation at the Standard Quantum Limit
Source: ACS Photonics. 2024 Jan 18;11(2):395–403. doi: 10.1021/acsphotonics.3c01159 (PMC10885206; doi:10.1021/acsphotonics.3c01159)
Supplement: Supplementary file 1 — ph3c01159_si_001.pdf [file ph3c01159_si_001.pdf]

Mid-Infrared Ring Interband Cascade Laser: Operation at the Standard Quantum Limit

Georg Marschick,[1, a)] Jacopo Pelini,[2, 3, a)] Tecla Gabbrielli,[4, 5] Francesco Cappelli,[4, 5] Robert Weih,[6] Hedwig Knötig,[1] Johannes Koeth,[6] Sven Höfling,[7] Paolo De Natale,[3, 4, 5, 8] Gottfried Strasser,[1] Simone Borri,[4, 5, 8] and Borislav Hinkov[1, 9]

[1)] *TU Wien – Institute of Solid State Electronics & Center for Micro- and Nanostructures, Gußhausstraße 25-25a – 1040 Vienna, Austria.*

[2)] *University of Naples Federico II, Corso Umberto I 40 – 80138 Napoli, Italy*

[3)] *CNR-INO – Istituto Nazionale di Ottica, Largo Fermi, 6 – 50125 Firenze FI, Italy*

[4)] *CNR-INO – Istituto Nazionale di Ottica, Via Carrara, 1 – 50019 Sesto Fiorentino FI, Italy*

[5)] *LENS – European Laboratory for Non-Linear Spectroscopy, Via Carrara, 1 – 50019 Sesto Fiorentino FI, Italy*

[6)] *Nanoplus Nanosystems and Technologies GmbH, Oberer Kirschberg 4 – 97218 Gerbrunn, Germany*

[7)] *Julius-Maximilians-Universität Würzburg – Physikalisches Institut, Lehrstuhl für Technische Physik, Am Hubland – 97074 Würzburg, Germany*

[8)] *INFN – Istituto Nazionale di Fisica Nucleare, Via Sansone, 1 – 50019 Sesto Fiorentino FI, Italy*

[9)] *current address: Silicon Austria Labs (SAL), Europastraße 12 – 9524 Villach, Austria*

(*Electronic mail: francesco.cappelli@ino.cnr.it)

(*Electronic mail: borislav.hinkov@tuwien.ac.at)

(Dated: 28 November 2023)

Appendix A: Tuning coefficient characterization

The estimation of the current-tuning coefficient gives insight into the relation between the emission wavelength of a laser and the applied bias current and was experimentally demonstrated to be pretty much linear[1,2]. For each optical spectrum shown in Fig. 2(b) the corresponding emission peak is fit to the following Gaussian function:

$$f(\lambda) = Ae^{-4\ln(2)(\lambda-\lambda_0)^2/\Delta\lambda^2} + f_0 \tag{A1}$$

where A is the amplitude, λ_0 the wavelength of the emission peak, $\Delta\lambda$ the full width at half maximum (FWHM) of the peak, and f_0 an offset. All of them are free parameters in the fit procedure. After converting the wavelength to the frequency domain by using the relation $c = f \cdot \lambda$, their linear regression is computed (see Fig. S1) to extract the current-tuning coefficient of our ring-ICL $\mathscr{T} = (903 \pm 2)$ MHz/mA.

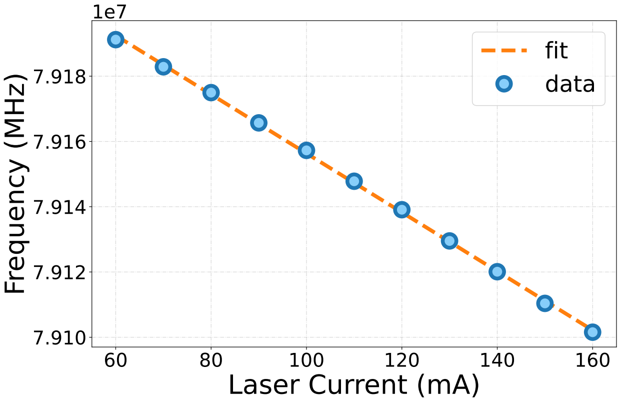

Fig. S 1. Current-Tuning coefficient characterization. The emission wavelength peaks are first converted into the frequency domain and then plotted as a function of the laser current (blue circles). The linear fit (dashed orange line) procedure allows us to estimate the tuning coefficient $\mathscr{T}$ = (903 ± 2) MHz/mA.

Appendix B: Detector Responsivities

The responsivity quantifies the efficiency of the detector for converting an input optical power signal into an output photocurrent (or photo-voltage). In the limit of linear response, it is given by:

$$\mathrm{R} = \eta_{\mathrm{qe}} \frac{\lambda \mathrm{e}}{\mathrm{hc}} \tag{B1}$$

where η_{qe} is the quantum efficiency, e the electron charge, λ the wavelength, h the Planck constant, and c the speed of light. In our work, we measure the responsivity of both detectors. At a fixed laser temperature of 16 °C we span the bias current from 60 mA to 150 mA with a step size of 10 mA. For each current value, we measure the emission power in front of the detector via a power meter (Thorlabs, PM400) and the pre-amplifier first-stage DC-output voltage. The acquired data is then fitted with a linear regression, and the estimated curve slope (in [V/W]) is divided by the transimpedance resistance (6 kΩ in our case) to obtain the

[a)] These authors equally contributed to this work.

detector responsivity in terms of the photocurrent.

As shown in Fig. S2, for a wavelength of $\lambda = 3.79\,\mu$m, the responsivity of Detector 1 and Detector 2 is $R_1 = (0.704\pm0.007)$ A/W and $R_2 = (0.659\pm0.008)$ A/W, respectively. From equation B1, it is possible to extract a quantum efficiency η_{qe} of (0.230±0.002) for the first detector and of (0.216±0.003) for the second one.

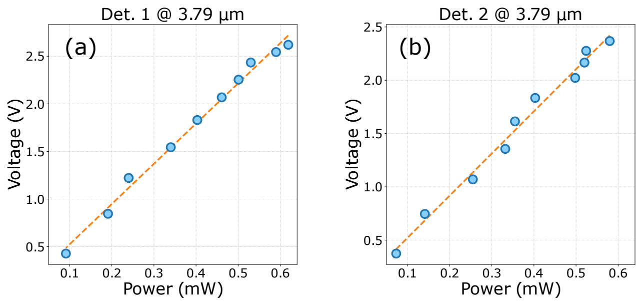

Fig. S 2. Responsivity of both detectors measured at a wavelength of $\lambda = 3.79\,\mu$m using the ring-ICL. A linear fit (dashed orange line) is computed to extract the following responsivities: (a) R1 = (0.704±0.007) A/W and (b) R2 = (0.659±0.008) A/W

.

Appendix C: Detector CMRR

The CMRR is calculated as the ratio between the INPSD of the sum and of the difference and quantitatively assets the noise rejection capability of our balanced detector[3]. To measure the maximum CMRR achievable with our setup, we modulate the laser current via the current driver module. The used modulation signal is a square wave of amplitude 0.8 V and with a carrier frequency of 100 kHz. The FFT of a square signal is characterized by all the odd harmonics generated starting from the main frequency. As shown in Fig. S3, the CMRR is calculated in this measurement at the harmonic frequencies of the used 100 kHz square wave. The experimental

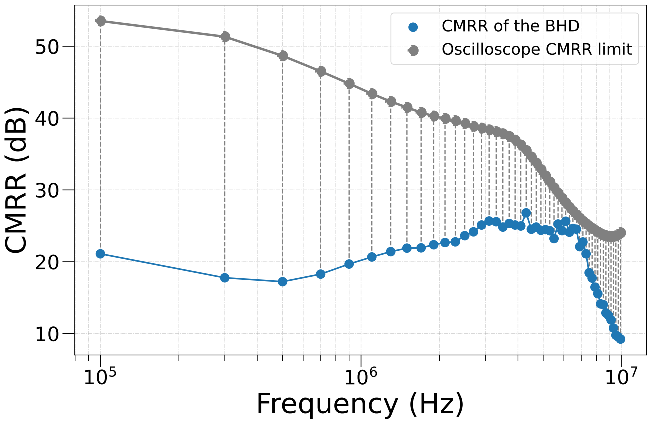

Fig. S 3. Common-Mode Rejection Ratio (CMRR) of the balanced detector (blue circles) and of the oscilloscope (grey circles).

data obtained with this procedure (blue dots) is compared to the maximum CMRR achievable with the oscilloscope (grey dots) when the same square signal is split via a t-connector and sent directly onto the two oscilloscope channels used for the measurements, as described in[3]. With our setup, we are able to achieve up to 25 dB (e.g. around 3 MHz). It is worth remarking that, the CMRR is a key parameter for differential measurements performed within the presented balanced detector[3], e.g. in case of application of this setup as a balanced homodyne detector. Indeed this parameter tells us how much noise can be tolerated and therefore suppressed when a differential measurement is performed.

Appendix D: INPSD analysis at different laser currents

Fig. S4 shows the INPSD analysis performed at 16 °C for different values of the current, i.e. (a) I = 80 mA, (b) I = 90 mA, (c) I = 100 mA, (d) I = 110 mA, (e) I = 120 mA, and (f) I = 130 mA. As expected, the INPSD of the sum and of the difference continuously move away from the detector background, since the shot noise level linearly increases with the photocurrent. In fact, at I = 80 mA (Fig. 4(a)), where the laser power is only 0.45 mW, the noise spectra slightly exceed the detector background, while at I = 130 mA (Fig. 4(f)), where the laser reaches an emission's power equal to 1.1 mW, the difference between the two levels is more appreciable, reaching a clearance of up to 6 dB at a Fourier frequency of around 1 MHz[3]. The ring-ICL intensity noise remains at the shot noise level within the whole range of investigation. In view of a concrete application of the assembled setup as a balanced homodyne detector where the tested ICL is used as the local oscillator, i.e. the radiation of reference used the probe the electric field properties of the light state understudy[3,4], the optimal working condition for the setup is the one with higher clearance, i.e. when the ring ICL is operated at I= 130 mA. This configuration guarantees indeed the highest signal-to-noise ratio within the assembled balanced detector[3].

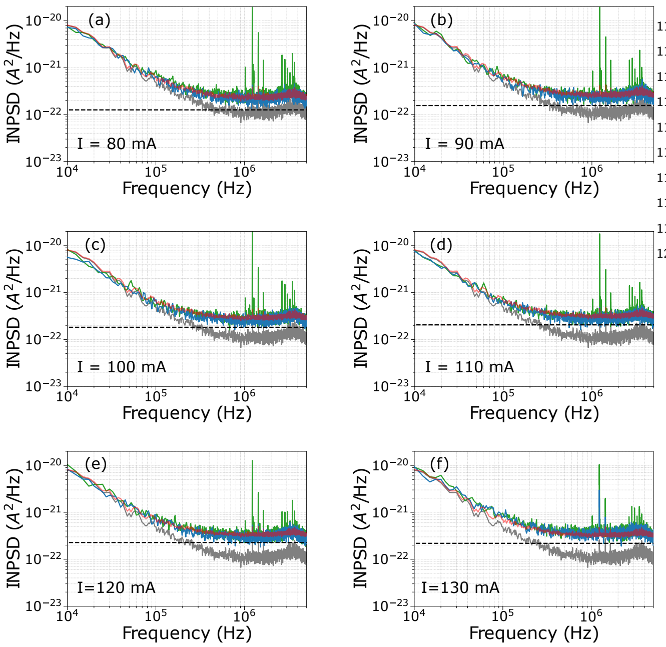

Fig. S 4. Ring-ICL INPSD analysis at a fixed temperature of 16 °C and for different laser bias currents: (a) I = 80 mA, (b) I = 90 mA, (c) I = 100 mA and (d) I = 110 mA, (e) I = 120 mA, (f) I = 130 mA. For each plot the theoretical shot-noise level is shown with a dashed black line, the detector background is depicted in gray, the sum of these two quantities is in red, the INPSD of the difference in blue, and the INPSD of the sum in green. As described in Fig. 4, also in these INPSD measurements the spurious noise around 1 MHz is present due to technical noise of a mass loop between the power supply of the detector and of the laser.

[1]Z. Du, G. Luo, Y. An, and J. Li, "Dynamic spectral characteristics measurement of dfb interband cascade laser under injection current tuning," Applied Physics Letters **109**, 011903 (2016).

[2]J. Hou, X. Chen, L. Wang, W. Chen, and N. Zhu, "A method of adjusting wavelengths of distributed feedback laser arrays by injection current tuning," IEEE Photonics Journal **4**, 2189–2195 (2012).

[3]T. Gabbrielli, F. Cappelli, N. Bruno, N. Corrias, S. Borri, P. De Natale, and A. Zavatta, "Mid-infrared homodyne balanced detector for quantum light characterization," Optics Express **29**, 14536–14547 (2021).

[4]R. Loudon, *The quantum theory of light* (OUP Oxford, 2000).
